# The Way Back from Tetraplegia or, Rare Neurological Manifestations of Eosinophil Granulomatosis with Polyangiitis

**DOI:** 10.3390/jcm14165652

**Published:** 2025-08-10

**Authors:** Yasamin Ranjbar, Tamás Árokszállási, Dorottya Szinay, Edit B. Nagy, Tünde Tarr, Melinda Nagy-Vincze

**Affiliations:** 1 Division of Immunology, Department of Internal Medicine, Faculty of Medicine, University of Debrecen, 4032 Debrecen, Hungary; 2 Department of Neurology, Faculty of Medicine, University of Debrecen, 4032 Debrecen, Hungary; 3 Doctoral School of Neuroscience, University of Debrecen, 4032 Debrecen, Hungary; 4 Gyula Petrányi Doctoral School of Allergy and Clinical Immunology, University of Debrecen, 4032 Debrecen, Hungary; 5 Division of Radiology and Imaging Science, Department of Medical Imaging, Faculty of Medicine, University of Debrecen, 4032 Debrecen, Hungary

**Keywords:** eosinophil granulomatosis with polyangiitis, central nervous system, tetraplegia, individualized treatment

## Abstract

Central nervous system (CNS) involvement is an extremely rare manifestation in eosinophilic granulomatosis with polyangiitis (EGPA), associated with a poor prognosis. Here we present a case of 50-year-old female patient with long-term asthma treatment who presented initially with extreme eosinophilia (56%) and severe progressive ascending paresis, similar to Guillain–Barré syndrome, leading to tetraplegia. After navigating through diagnostic mazes, the diagnosis of EGPA was established based on eosinophilia, myeloperoxidase anti-neutrophil cytoplasmic antibody (MPO-ANCA) positivity, asthma, eosinophil granulomatosis in the gastrointestinal tract, and severe peripheral nervous system involvement, complicated with rare central nervous granulomas and ischemia. With combined immunosuppressive and immunomodulatory treatment including high-dose corticosteroids, rituximab and intravenous immunoglobulin along with symptomatic treatment and planned rehabilitation over 6 months, our patient recovered gradually from tetraplegia and adverse events such as severe infections and osteoporotic fractures. Now, from a 2-year perspective, we can conclude a successful treatment leading to decrease in all of her symptoms. Due to persistent eosinophilia after steroid tapering, she was switched to mepolizumab maintenance treatment and demonstrated continuous improvement of motor and sensory functions. Thanks to periodically repeated rehabilitation, she became self-sufficient and returned to her previous job. Our case highlights that EGPA patients should be treated in a center of expertise due to the rarity of the disease and complexity of diagnosis and treatment. Careful multidisciplinary cooperation, the huge effort of the patient, and a supportive environment can show a way back from immune-mediated tetraplegia.

## 1. Introduction

Eosinophilic granulomatosis with polyangiitis (EGPA), previously known as Churg–Strauss syndrome, is a rare systemic immune-mediated disorder characterized by eosinophilia, anti-neutrophil cytoplasmic antibody (ANCA)-associated vasculitis (AAV) of small and medium-sized vessels, and pulmonary symptoms [1,2,3]. Its prevalence ranges from 1.7 to 13 cases/million inhabitants. ANCA positivity ranges from 30 to 70% of EGPA patients but is usually less frequently observed than in other AAVs [1,2,3,4,5,6,7,8]. EGPA primarily affects the respiratory tract, lungs, peripheral nervous system, heart, gastrointestinal tract, and skin, but CNS involvement is rarely seen [4]. Some investigators hypothesize an association between EGPA onset and medications such as leukotriene receptor antagonists. However, this is more due to unmasking the underlying disease rather than directly causing EGPA [9]. Several studies considered Human Leukocyte Antigen (HLA-DRB1 and HLA-DRB4) genes as possible susceptibility markers for EGPA [9]. The pathogenesis of EGPA has yet to be completely understood. It is considered to be primarily T-cell mediated vascular injury. CD4-positive T lymphocytes secrete gamma interferon, which promotes granulomatous inflammation. Interleukins (IL-4, IL-5, and IL-13) activate eosinophils, thus releasing proteins that cause damage to endothelial cells. This damage leads to the release of eotaxin-3 from endothelial cells, which attracts more eosinophils [9]. Based on previous data, the EGPA disease course has three different phases. The initial prodromal phase typically lasts months to years and includes arthralgia, myalgia, and fever. The eosinophilic phase is characterized by peripheral eosinophilia and the involvement of organs, including the lung, the heart, and the gastrointestinal tract. Finally, the vasculitis phase of EGPA is characterized by medium/small vessel vasculitis, sensorimotor peripheral neuropathy, and stroke [10]. Anti-myeloperoxidase (MPO)-ANCA-positive patients have a more vasculitis phenotype with peripheral neuropathy, purpura, renal involvement, and biopsy-proven vasculitis; meanwhile, MPO-ANCA-negative patients have higher risk of cardiac involvement [11].

The frequency of CNS manifestations varies from 5% to 13% [3,5,6]. Ischemic lesions, intracranial hemorrhage, granulomas of the spinal cord and medulla oblongata, cranial nerve palsies, and loss of visual acuity due to optic neuritis have been reported [3,4,5,6]. Liu et al. reported that more than 50% of patients with CNS involvement also presented gastrointestinal symptoms and fever [5]. Meanwhile, peripheral neuropathy is considered as a relatively common symptom of EGPA, which occurs in 46–55% of patients [5,6]. The pathophysiology of CNS involvement in EGPA is not completely defined and is probably more complex than in other AAV because the disease frequently associates vasculitis, blood, and tissue eosinophilia [6]. Peripheral nerve involvement is caused by axonal degeneration due to ischemia that is secondary to damage of the vasa nervorum. Clinical characteristics are painful paresthesia, numbness, and, later, motor impairment and muscular atrophy [10]. Table 1 summarizes previously reported neurological manifestations of EGPA.

During the diagnostic procedures, laboratory and imaging techniques are used to evaluate the organ involvement as follows: computed tomography (CT) for lung involvement, magnetic resonance imaging (MRI) and electroneurography (ENG) for neurological manifestations, endoscopies in case of gastrointestinal symptoms and echocardiography, cardiac MRI for myocardial involvement. The Birmingham vasculitis score (BVAS) and other scoring systems like the five factor score (FFS) are useful to evaluate disease activity and therapeutic response [11,12,13]. Indirect immunofluorescent techniques and enzyme-linked immunosorbent assay (ELISA) [12] can prove the presence of antibodies.

Data regarding therapeutic management and outcomes of CNS involvement are scarce. In addition, central nervous system involvement in EGPA represents a huge therapeutic challenge, since it is responsible for long-term sequelae and death, especially in those with intracerebral hemorrhages [6]. Previous treatment guidelines have suggested the use of corticosteroids combined with cyclophosphamide (CYC) in the case of life- and/or organ-threatening disease manifestations (i.e., heart, gastrointestinal, central nervous system, severe peripheral neuropathy, severe ocular disease, alveolar hemorrhage, and/or glomerulonephritis). Methylprednisolone pulse (0.5–1 g/day for 3–5 days), high-dose glucocorticoids (1–2 mg/kg/day), and low-dose regimens also were presented (<0.5 mg/kg/day) as options in the case of mild clinical symptoms. Intravenous immunoglobulin (IVIg) or plasma exchange therapy could be considered in therapy refractory cases and intrathecal injections of dexamethasone (10 mg each time) and methotrexate (MTX, 10 mg each time) were used in case of CNS involvement [5,6,7,8,9,12]. The latest guidelines prefer aggressive induction treatment, such as cyclophosphamide or rituximab, in combination with glucocorticoids, followed by maintenance therapy (with azathioprine or methotrexate). IVIg remains a rescue treatment in the case of infective complications [13]. The important role of eosinophils in EGPA and recent development of effective agents to treat other eosinophil-related diseases (e.g., asthma, hypereosinophilic syndrome) have created new therapeutic possibilities such as anti-interleukin-5 agent (mepolizumab). Reslizumab and the IL-5 receptor antagonist benralizumab are both promising, as IL-5 is the major cytokine responsible for eosinophil activation, chemo attraction, and survival. IL-4 and IL-13 are major cytokines for helper T cells and eosinophil activation. Agents such as dupilumab, pitrakinra, and lebrikizumab block the IL-4 receptor or the circulating IL-13. Ongoing trials in asthma may open up new opportunities for EGPA treatment. Interferon alpha may be reserved as a third-line treatment of therapy refractory cases [12,13].

## 2. Case Presentation

**Figure 1:**
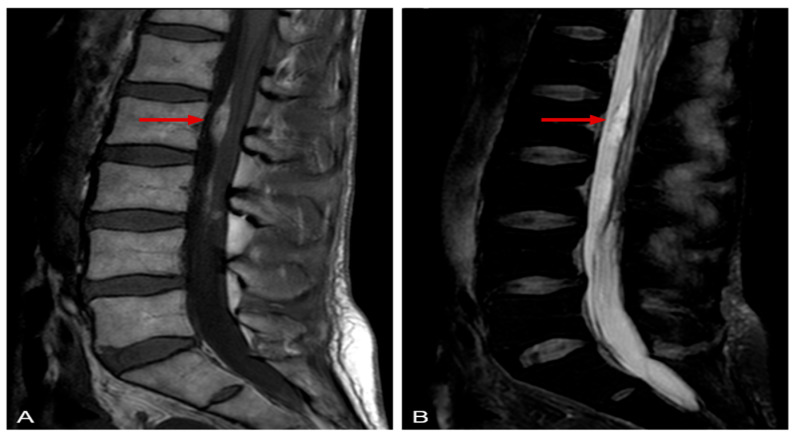
Lumbar spine magnetic resonance imaging (MRI). (**A**) T1-weighted sagittal image shows a band-like hyperintense lesion spanning the levels of lumbar vertebrae L1 to L3, without contrast administration. (**B**) T2-weighted fat-suppressed sagittal image demonstrates a similar band-like intradural hyperintense lesion, indicating the absence of fat content.

Our patient is a 50-year-old female with a history of bronchial asthma previously treated with inhaled beta-2 agonist, antihistamine, and inhaled and/or oral low-dose corticosteroids. She was referred to our clinic (a tertiary center in clinical immunology) due to muscle pain, muscle weakness, and numbness in the left upper limb and right lower limb which started after an upper-airway infection. MRI of the cervical spine showed protrusion of the intervertebral disks that was not consistent with neurological symptoms. During the first period of diagnostic procedures, new symptoms such as vomiting and abdominal pain were presented. Chest X-ray and brain CT were negative but further investigations revealed extreme eosinophilia (56%) and a suspicion of cholelithiasis with cholecystitis. Due to diffuse persistent abdominal pain, a laparoscopic cholecystectomy was performed. The operation was converted to an exploratory laparotomy due to signs of circulatory disturbance and edema in the wall of the small intestine. Significant intraabdominal findings, besides the abovementioned, were several small (1 mm) nodules along the mesenteric vessels. Vasculitis was suspected. Unfortunately, histology was partially assessable, and only inflammatory cellular infiltration was reported. Meanwhile, the neurological symptoms worsened. ENG examinations revealed peripheral sensory and motor polyneuropathy. Elevated protein, albumin level, and elevated leukocyte count were reported after analysis of cerebrospinal fluid. These signs were initially misdiagnosed as atypical Guillain–Barré syndrome. Laboratory findings such as monoclonal gammopathy of unknown significance (MGUS) and thrombocytosis also disturbed the overall picture. Bone marrow biopsy and genetic tests excluded essential thrombocytosis. The neurological symptoms progressed to tetraparesis, areflexia, and a lack of pyramidal signs, as well as tactile and thermal hypesthesia at the area of the C4 dermatome. Repeated ENG examination reported severe progressive, axonal dominant sensorimotor polyneuropathy, electromyography (EMG) revealed acute denervation of muscles (right anterior tibial, left vastus lateralis, and right deltoid). Lumbar MRI showed abnormalities consistent with EGPA (spindle-shaped granulomatous lesions along L1–L3) (Figure 1).

**Figure 2:**
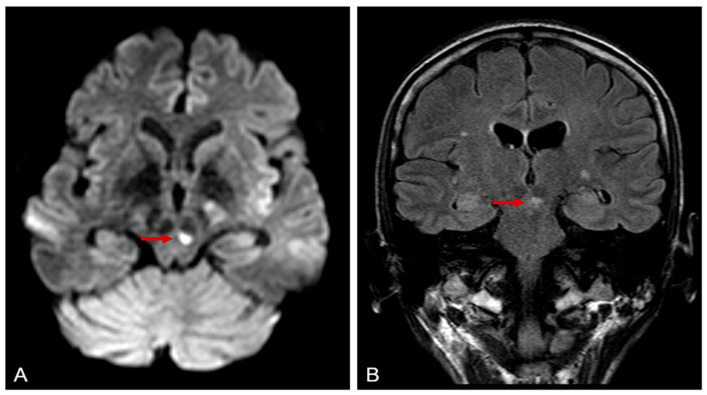
Brain magnetic resonance imaging (MRI). (**A**) Diffusion-weighted axial image (DWI) and Fluid Attenuated Inversion Recovery (FLAIR) (**B**) coronal image demonstrate hyperintense lesion in the mesencephalon, consistent with an acute ischemic lesion.

Based on asthma, eosinophilia greater than 10%, mononeuropathy (including multiplex), polyneuropathy, mesenteric and spinal cord granulomas, and MPO positivity, the diagnosis of EGPA was established. After excluding atypical bacterial and viral infections as well as malignancy and parallel to treatment of nosocomial infections, we started pulse steroid (500 mg of methylprednisolone for three consecutive days) treatment. Along with steroid tapering, use of intravenous immunoglobulin (2 g/kg every month) helped the patient through several nosocomial infections (pneumonia, peritonitis, sinusitis, and urinary tract infection). Another challenge was the treatment of malabsorption and cessation of weight, especially muscle loss. Intermittent parenteral feeding and vitamin and protein supplementation, with a personalized exercise program, was effective. Although laboratory results improved, extreme eosinophilia seems persistent and repeated ENG showed worsening in axonal and motor polyneuropathy as well as mononeuritis. We started rituximab (375 mg/m2 per week for 4 weeks) as an induction treatment according to European Alliance of Associations for Rheumatology (EULAR) guidelines [13]. Unfortunately, the third and fourth dosage needed to be delayed due to another nosocomial and severe coronavirus (COVID-19) infection. This viral adverse event reactivated the disease, and a new ischemic lesion was reported in mesencephalon (Figure 2), with increasing MPO titers.

**Figure 3:**
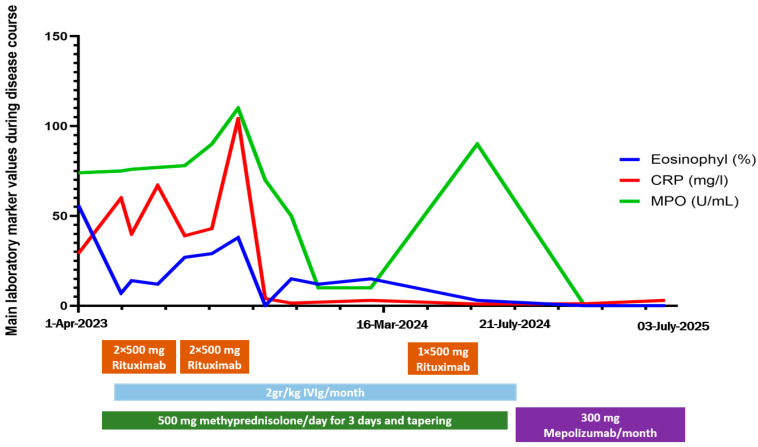
Changes in most important lab values (eosinophilia (%), C-reactive protein (CRP; U/L), and anti-myeloperoxidase (MPO) antibody titer (U/mL) and treatment regimen in time.

Simultaneously with the COVID-19 infection, inferobasal hypokinesis appeared on the echocardiography. The careful balance of rituximab and IVIg treatment, alongside steroid tapering and supportive care, led to partial remission after 6 months of in-patient treatment. After rehabilitation programs, due to persistent eosinophilia, we decided to change the planned rituximab maintenance therapy to 300 mg/months mepolizumab, also considering the abovementioned EULAR guidelines [13]. Due to the high dose methylprednisolone therapy, despite all preventive effort (vitamin D and calcium supplementation, physiotherapy) patient developed osteoporosis and suffered vertebral compressions. We added teriparatide to her basic treatment and used a special auxiliary device and observed an improvement in spinal stability. Nowadays, she can walk without any aid; she is self-sufficient and has returned to her previous job. In the last 2 years, the patient has been constantly improving, without relapses. Figure 3 presents changes in laboratory markers and therapeutic regimen.

## 3. Discussion

Our case presents rare and severe combined neurological manifestations of EGPA. Vasculitis-associated manifestations were mesenteric granulomas, mononeuritis, polyneuropathy, CNS ischemia, and granuloma. The eosinophil-mediated component was the asthma. Its overlapping features with other vasculitis or eosinophilic diseases, and the wide and heterogeneous range of clinical manifestations resulted in a delay to diagnosis. Eosinophilic and vasculitis manifestations are often intermingled. Eosinophils may be a component of vascular and perivascular infiltrates and a complete distinction between eosinophilic and vasculitis manifestations cannot be always established. Acute progressive sensory–motor polyneuropathy, which initially mimicked Guillain–Barré syndrome, also lead to diagnostic difficulty. Neuropathy also may have a vasculitis origin and/or an eosinophil-derived neurotoxicity component [14]. The uniqueness of this case lies in the rare granulomatous CNS involvement intertwined with cerebrovascular symptoms, peripheral neuropathy, and mononeuritis. So far, seven cases have been published where EGPA presented as polyneuropathy mimicking Guillain–Barré syndrome, but only one of them had multiorgan involvement and none of them were associated with CNS involvement. Five patients reported improvement besides combine immunosuppressive treatment. Two patients had a worse outcome. The majority of these cases were associated with ANCA positivity, as in our case [15,16].

A limitation of the study is the absence of histological support of our diagnosis and the lack of information from the enteral biopsy. Although based on the classification criteria assessed by American College of Rheumatology (ACR) and EULAR [17], the patient had 9 points, meaning that vasculitis could be classified as EGPA even without a biopsy result.

Our case also presents a long and hard clinical care journey, with several therapeutic difficulties, all of which could have led to a fatal outcome. In a French multicenter study, 11 of 26 patients (42%) patients with CNS involvement died within 3 years from diagnosis [6]. A balance between immunosuppressive treatment and nosocomial infections is usually instable in such a severe case. The availability of IVIg treatment fundamentally determined the end of our case report. Generally, overall survival of EGPA seems good. A French vasculitis study group published the outcome data of a cohort of 118 patients with EGPA [8]. Overall survival reached 90% at 7 years, regardless of baseline severity. Older age (≥65 years) was the only factor associated with a higher risk of death. The relapse rate was higher for patients with anti-myeloperoxidase antibody positivity. A larger American cohort with 354 patients and with a median follow-up of 7 years showed 4% mortality [18].

A retrospective cohort study from the US demonstrated that health care costs are approximately 2.5-fold higher in patients with EGPA than in patients with asthma who have similar demographic characteristics. Patients with EGPA also require more health care utilization and systemic corticosteroid use than patients with asthma. In addition, more than one third of patients with EGPA experienced relapses [19]. Chinese cohorts with CNS involvement also reported 34% of relapses during steroid reduction [5]. These results highlight the unmet need of new therapies that make it possible to reach stable and complete remission as well as steroid tapering after induction therapy. New therapies like rituximab, mepolizumab, or other biologics also can lead to less common or less severe comorbidities (such as osteoporosis, hypertension, and diabetes) with a steroid-free maintenance treatment. Data from the Spanish registry of Systemic Vasculitis (REVAS) also showed improved outcomes, with significant decrease in mortality and treatment-related morbidity in patients diagnosed after 2000. Improving outcome data also was related to the implementation of less toxic regimens adapted to the disease activity and stage, and a significant reduction in glucocorticoid dose [20].

## 4. Conclusions

Our findings indicate that EGPA should be considered as a differential diagnosis of rapidly progressing polyneuropathy, with or without CNS involvement, at a young age, especially in the presence of eosinophilia. These patients need to be managed in multidisciplinary collaboration with or in centers with established expertise in the management of vasculitis. Patients with peripheral nerve involvement and motor deficit(s) should also be routinely referred to a physiotherapist. Careful multidisciplinary cooperation, individualized, targeted therapy, the huge effort of the patient, and a supportive environment can provide the way back from immune-mediated tetraplegia. New therapeutic options may help achieve remission, steroid sparing, and reduce comorbidities.

## Figures and Tables

**Table 1:** Neurological manifestations of EGPA based on the cited literature.

Neurological Manifestations of EGPA	
Central Nervous System	Peripheral Nerves
Ischemic lesions	Mononeuritis multiplex
Intracranial hemorrhage Epidural bleed Subdural bleed Subarachnoid bleed Intracerebral hemorrhage	Polyneuropathy
Granulomas on the spinal cord	Sensorineural hearing loss
Encephalopathy	Autonomic neuropathies
Epilepsy	
Cranial nerve palsies	
Hydrocephalus	
Optic neuritis	

## Data Availability

The data presented in this study are available on request from the corresponding author due to privacy.

## Abbreviations

AAV	ANCA-associated vasculitis
ACR	American College of Rheumatology
ANCA	anti-neutrophil cytoplasmic antibody
BVAS	Birmingham vasculitis score
CNS	central nervous system
CYC	cyclophosphamide
CT	computed tomography
COVID-19	coronavirus
ENG	electroneurography
EMG	electromyography
ELISA	enzyme-linked immunosorbent assay
EULAR	European Alliance of Associations for Rheumatology
EGPA	eosinophilic granulomatosis with polyangiitis
FFS	five factor score
IL	interleukin
IVIg	intravenous immunoglobulin
MRI	magnetic resonance imaging
MTX	methotrexate
MGUS	monoclonal gammopathy of unknown significance
MPO	anti-myeloperoxidase
REVAS	Spanish registry of Systemic Vasculitis
Th	helper T cells
